# Why Are the Voluntary Hospital Managers Inactive?

**Published:** 1912-11-30

**Authors:** Walter Alvey


### The Editor's Letter-Box.

### Why are the Voluntary Hospital Managers Inactive?

*To the Editor of* THE HOSPITAL.

SIR,—In your issue of this date you ask me to explain what is the cause of the present inaction on the part of hospitals in regard to the Insurance Act. It is, in my opinion, twofold. In the first place, hospitals are waiting upon the decision of the doctors, for the question of their own policy depends intimately upon the policy to be adopted by the medical profession. But, even when this difficulty is removed, I think there will still be an absence of concerted action, for the simple reason that no machinery exists to produce it.

As I said in the speech from which you quote, what we want is some representative body whose function it shall be to consider, as they arise, questions (such as the Insurance Act) affecting the hospitals as a whole, and to formulate a policy in regard to them. Until some such body—some central advisory council—recognised by the hospitals themselves, comes into being it is idle to expect (except through special and laboriously arranged conferences) common action upon any of those matters that touch the interests of all the hospitals and seem likely to become more numerous as time goes on.—Yours faithfully,

WALTER ALVEY.

November 23.

*** We note, and it is significant, that Mr. Walter Alvey does not mention the British Hospitals Association, the responsibility and inactivity of which we refer to in another column. It is clearly the special work of the British Hospitals Association, if it is a live organisation and to be of any value to the voluntary hospital system, that it should wake up and be doing at this crisis. The work which Mr. Alvey refers to is clearly the work of this Association, or it will not be easy to justify its continued existence. Should the British Hospitals Association fail to do the work it was formed to accomplish, our correspondent's letter means others will speedily be forthcoming to accomplish all that Mr. Alvey properly insists requires to be done. Throughout the hospital world everybody is asking: "Why this strange inaction on the part of the British Hospitals Association?"—ED. THE HOSPITAL.

